# Phenological Variation in *Ambrosia artemisiifolia* L. Facilitates Near Future Establishment at Northern Latitudes

**DOI:** 10.1371/journal.pone.0166510

**Published:** 2016-11-15

**Authors:** Romain Scalone, Andreas Lemke, Edita Štefanić, Anna-Karin Kolseth, Sanda Rašić, Lars Andersson

**Affiliations:** 1 Swedish University of Agricultural Sciences, Department of Crop Production Ecology, Uppsala, Sweden; 2 Technische Universität Berlin, Department of Ecology, Plant Ecology and Ecosystem Science, Berlin, Germany; 3 University of Josip Juraj Strossmayer, Faculty of Agriculture, Osijek, Croatia; INRA - University of Bordeaux, FRANCE

## Abstract

The invasive weed *Ambrosia artemisiifolia* (common ragweed) constitutes a great threat to public health and agriculture in large areas of the globe. Climate change, characterized by higher temperatures and prolonged vegetation periods, could increase the risk of establishment in northern Europe in the future. However, as the species is a short-day plant that requires long nights to induce bloom formation, it might still fail to produce mature seeds before the onset of winter in areas at northern latitudes characterized by short summer nights. To survey the genetic variation in flowering time and study the effect of latitudinal origin on this trait, a reciprocal common garden experiment, including eleven populations of *A*. *artemisiifolia* from Europe and North America, was conducted. The experiment was conducted both outside the range limit of the species, in Sweden and within its invaded range, in Croatia. Our main hypothesis was that the photoperiodic-thermal requirements of *A*. *artemisiifolia* constitute a barrier for reproduction at northern latitudes and, thus, halts the northern range shift despite expected climate change. Results revealed the presence of a north-south gradient in flowering time at both garden sites, indicating that certain European populations are pre-adapted to photoperiodic and thermal conditions at latitudes up to, at least, 60° N. This was confirmed by phenological recordings performed in a region close to the northern range limit, the north of Germany. Thus, we conclude that there exists a high risk for establishment and spread of *A*. *artemisiifolia* in FennoScandinavia in the near future. The range shift might occur independently of climate change, but would be accelerated by it.

## Introduction

Range shifts of plants are expected as a consequence of climate change, characterized by higher temperatures, changes in precipitation patterns and prolonged vegetation periods [1]. Distribution beyond the current range limit might occur gradually as the climate changes and new areas come within the species niche limit [2]. Several attempts have been made to predict the range shift of southerly species to northern latitudes [1, 3, 4], based on assumptions of a change in climate. The geographic shift of species over time in response to contemporary climate change has been estimated to 1.69 km year^-1^ towards northern latitudes, and is in general sufficient to track temperature changes [5]. It has, however, been questioned whether this conclusion is valid for lowland terrestrial plants [6, 7]. A British study [8] revealed no clear fingerprint of climate change on poleward movement of plants, and an expected lag in distribution at the leading edge was suggested. A lag phase is commonly observed in invasive species, which makes it difficult to predict the timing of the different processes related to invasion [9]. The lag phase is poorly understood but it may be partly explained as the time needed for the species to evolve and adapt to the new habitat [10]. Consequently, Whitney & Gabler [11] concluded that evolutionary potential should be incorporated in the assessment of the invasiveness of a species, and mentioned short generation time as one of ten key traits. One reason for this could be a requirement for changes in flowering time since many plant species (i.e. short-day plants) require long nights for induction of flowering [12]. Thus, for northern range expansion to occur in short-day species an adaptation to new photoperiodic-thermal conditions (i.e. photoperiod, temperature and their interaction) is required, as has been observed in the short-day grass *Microstegium vinimeum* [13]. Several studies have reported earlier flowering in populations sampled at the northern range margins compared to the southerly counterparts [14–16].

The invasive short-day plant *Ambrosia artemisiifolia* L. (common ragweed) is considered to be one of the most noxious plants in Europe [17]. This ranking is based both on its negative effects on public health and its reductive impacts on the yields of several major crops. This dramatic situation has led to international cooperation among scientists to control the species in a sustainable way (COST Action FA1203 “Sustainable Management of *A*. *artemisiifolia* in Europe” [18]). Health problems caused by *A*. *artemisiifolia* include dermatitis and eczema after direct skin contact [19], and respiratory problems and asthma due to the release of abundant quantities of highly allergenic pollen grains [3]. Moreover, the late flowering time due to its short-day plant trait extends the period of hay fever for allergy sufferers until late autumn [20, 21]. The pollen grain production from a single *A*. *artemisiifolia* plant has been estimated to several billion per season [22] and its dispersal via wind to several hundred kilometres [23, 24]. In addition to impact on human health *A*. *artemisiifolia* is recognized as a highly competitive weed, with high infestation of row crops such as sunflower and maize, in cropping systems in Western, Central and Eastern Europe [25, 26]. In Hungary, for example, 70% of agricultural fields are reported to be infested [21].

**Fig 1:**
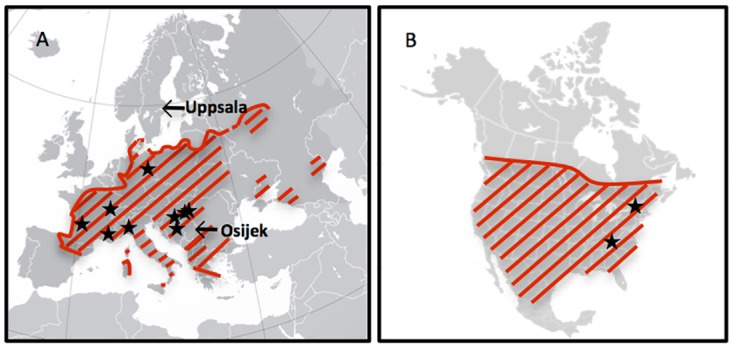
Distribution of *Ambrosia artemisiifolia* in A) Europe (modified from Smith et al. 2013 [32]) and B) North America (modified from Genton et al. 2005 [33]). Stars mark the location of populations sampled for the experiment, and arrows indicate the location of the common garden sites in Uppsala and Osijek, respectively. Attribute, Europe map: Ssolbergj - https://commons.wikimedia.org/w/index.php?curid=4203715. Attribute, North America map: Alan Rockefeller - https://commons.wikimedia.org/wiki/File%3ANorth_america_blank_range_map.png.

Originating from North America, with a known distribution from latitude 31°N to 52°N, several genotypic studies have shown that the successful worldwide invasion and range expansion of *A*. *artemisiifolia* is the result of multiple introductions from different regions of the native range [25, 27–31]. It was first spread to Europe in the middle of the 19^th^ century and has since been repeatedly introduced to Europe. The main area of infestation is Central Europe, but it is now established in large parts of the continent, with a northern range limit situated south of the Scandinavian countries and the UK, and the southern range limit in mid Italy, the Iberian Peninsula and Greece [1, 4] (Fig 1). With climate change, the northern limit, based on environmental factors, of common ragweed range is the one most likely to be extended.

The species has been reported to require a minimum night length of eight hours to induce flowering [34]. Night length, defined as the time difference between sunset and sunrise, never falls below eight hours in Budapest (latitude 47°30´ N), but does not exceed eight hours until mid-August in Stockholm (latitude 59°51´ N) or mid-July in Berlin (latitude 52°31´ N) [35]. Photoperiodic conditions in northern Europe might thus delay the onset of flowering until autumn. Indeed, according to Artportalen, the Swedish Species Information Centre [36], the earliest day reported for flowering of *A*. *artemisiifolia* between 2004 and 2013 was 21 August (day length: 15 h 4 min. / night length: 8 h 56 min.). This appears to be too late to enable production of viable seeds before plants are killed by frost, which could explain why there are, to our knowledge, no confirmed established populations in Sweden. Since the photoperiod is a stable factor, unaffected by climate change [12], it might serve as a barrier against establishment of *A*. *artemisiifolia* in the north of Europe despite improved climatic conditions. There are, however, two factors, which might overrule the preventative effect of a short summer photoperiod. First, there may exist populations at the northern range margin of *A*. *artemisiifolia*, which are pre-adapted to northern photoperiodic-thermal conditions. These populations could gradually expand their range limit, aided by on-going changes in climate. Secondly, the commercial trade in birdseed infested with seeds of *A*. *artemisiifolia* is the main source of introduction in Scandinavia, which makes private gardens and municipal soil deposits common casual habitats. Continuous introduction of *A*. *artemisiifolia* seeds in Scandinavia might contribute to the process of selection of early-flowering individuals, which would subsequently enable the establishment of viable populations. Although gene flow may counteract local adaptation, multiple introductions have been shown to enhance invasive success by introducing novel alleles and increasing genetic variation [37]. When a species has similar ecological and climatic niches in both invasive and domestic populations, invasions typically fail without pre-adaptation and the rate of migration of suitable alleles along the selective gradient becomes crucial for adaptation [38]. Under these circumstances seeds of pre-adapted genotypes hitchhiking with contaminated birdseed can also be important. All scenarios are in line with conclusions by Clements & DiTommaso [39] who argued that evolution of invasive weeds might increase the range shift more than forecasted and faster than the process of climate change. For the two scenarios of range shift to be realistic for *A*. *artemisiifolia*, the photoperiodic-thermal requirements for flowering should contain genetic variation. Recent work using common garden experiments and genomic tools has confirmed a differentiation of life-history traits between native and introduced populations [40]. The highly diverse gene pool present in Europe has promoted a rapid evolution and adaptation of *A*. *artemisiifolia* to different environments, e.g. field, roadside, river-side and wasteland [41]. In addition, the presence of a north-south gradient in flowering time within introduced European populations from latitude 54° N to 44° N was recently shown in a common garden experiment located within the invaded area [16].

The aim of this study was to explore variation in phenology within *A*. *artemisiifolia* populations of different origins and test whether there exists a latitudinal cline in flowering time. Common garden experiments have a long tradition in evolutionary ecology, and are well suited for studying the genetic differentiation among genotypes. Growing different genotypes of a species in a common environment makes it possible to attribute phenotypic variation to either genetic or environmental factors [42]. A reciprocal common garden experiment was conducted at one site in the middle of the invaded area (Osijek, Croatia; latitude 45°N), and at one site beyond the range limit (Uppsala, Sweden; latitude 59°N). The experiment was complemented with phenological observations at several field sites of populations located in the northern part of the invaded range (Germany; latitude 51–52°N). We hypothesized that i) there exists a north-south gradient in flowering time among populations, with plants from northern populations flowering earlier, and ii) the gradient shows the same pattern at both sites, but is more distinct at the northern site due to non-optimal photoperiodic-thermal conditions beyond the range limit. The absence of a north-south gradient would indicate that there is no local adaptation to photoperiodic-thermal conditions, but rather that the variation in flowering time is based on phenotypic plasticity.

## Materials and Methods

### Common garden experiment

A total of eleven populations of *Ambrosia artemisiifolia* were cultivated in a reciprocal common garden experiment, with one garden site located beyond the range limit, at a high latitude (Uppsala, Sweden: N 59° 48´ 55”, E 17° 38´ 47”) and the other within the invaded European range (Osijek, Croatia: N 45° 31´ 16”, E 18° 40´ 54”). Common garden experiments were conducted on land owned by the Swedish University of Agricultural Sciences and University of the Josip Juraj Strossmayer, respectively, and permissions for the experiments were granted by the universities. Seeds of *A*. *artemisiifolia* were collected in 2010 and 2011 from nine introduced populations in five European countries and two native populations from North America (Fig 1, Table 1). No special permission was needed for seed collection and field studies since they did not involve endangered or protected species. The eleven populations were selected to represent the latitudinal variation from latitude 38°N to latitude 51°N. Seeds from at least ten individual plants were collected per population. A population was defined as a set of individuals growing in one field with at least 7.5 km between sampled fields. Seed weight was calculated as the mean weight of 5 x 50 seeds. Pretreatment of seeds and handling of seedlings were conducted according to the same experimental protocol in both Sweden and Croatia, as follows. A total of ca. 100 seeds per population were placed on moist paper in Petri dishes at 5°C for stratification. Subsequently, the Petri dishes were placed in a growth chamber with a temperature regime of 15°C at night (no light) for 8 hours and 27°C at day (light intensity: 50 μmol m^-2^ s^-1^) for 16 hours to induce seed germination. When around 50 seedlings per population had been produced, seedlings were planted in plastic trays with individual wells (5 cm diameter, 6 cm depth). Each tray was assigned to a specific population, and the soil used to fill the trays was identical to that used for the garden phase. Soils with similar composition were used at both sites (fertilized peat substrate; Hasselfors Garden S-jord in Uppsala and Klasmann TS1 in Osijek). The position of the trays was randomized once a day. The seedlings were grown in a closed greenhouse (20°C) until the four-leaf stage, which occurred ca. 3 weeks after germination. Subsequently, ten four-leaf seedlings of each of the eleven populations, as similar in size as possible, were selected and transplanted in pots (5.5 L, top Ø 19.5 cm, height 25.5 cm; Soparco, Condé-sur-Huisne, France) filled with 5 L soil. The pots were placed outdoors in the garden in a completely randomized design with 1 m between each pot. The plants were watered throughout the experimental period to avoid drought and fertilized three times with in total 0.1418 g N per pot, corresponding to 50 kg N ha^-1^. The common garden experiment in Uppsala started with the transplantation of seedlings outdoors on 15 June and ended on 16 October, shortly before the first frost. In Sweden, *A*. *artemisiifolia* plants have been observed already in May [36] and the settings of the germination for the common garden experiment (end of May) were chosen based on these observations. Hence, early flowering by the German population at the Uppsala site is more likely to be a consequence of local adaptation rather than wrong timing of the garden experiment. The experiment at Osijek started on 16 June and finished on 11 September, when all populations had produced both male and female flowers.

**Table 1:** Details of the origin of *Ambrosia artemisiifolia* populations grown in a reciprocal common garden experiment.

Population	Country	Latitude, Longitude	Year of collection	Habitat	Seed contributor
Drebkau	Germany	N51°38´21", E14°11´50"	2011	fallow field	U. Starfinger
Martonvásár	Hungary	N47°20´37", E18°50´31"	2011	field	P. Bonis
Baracska	Hungary	N47°18´03", E18°45´51"	2011	field	P. Bonis
Pluvet	France	N47°11´18", E05°15´01"	2011	maize field	B. Chauvel
Kaposvár	Hungary	N46°22´12", E17°51´17"	2011	maize field	G. Kazinczi
Besate	Italy	N45°18´25", E08°58´21"	2011	road edge	M. Bonini
St Clothilde	Canada	N45°10´03", E73°40´50"	2011	field edge	D. Benoit
Bassens	France	N44°54´04", E00°31´58"	2010	waste land	B. Laitung
Pribinic	Bosnia & Herzegovina	N44°35´59", E17°49´40"	2011	waste land	B. Vuckovic-Kelevic
Dions	France	N43°56´03", E04°18´22"	2010	waste land	B. Laitung
Lexington	USA	N38°01´00", E84°33´10"	2010	old pasture	C. & J. Baskin

Ten individuals from each population were grown in Uppsala, Sweden (N 59° 48´ 55”, E 17° 38´ 47”) and in Osijek, Croatia (N 45° 31´ 16”, E 18° 40´ 54”). Latitude and longitude refer to seed sampling site, and habitat refers to vegetation type at seed sampling site.

All plants were visually inspected at least twice a week to record the different phenological stages: i) first male flower (i.e. the first emergence of anthers outside one single male flower, usually located in the terminal male inflorescence), and ii) first female flower (i.e. appearance of pistils outside one single female flower). After the first release of pollen grains from an individual plant, male inflorescences of the plants at the garden site at Uppsala were cut continuously until the end of the experiment. This precautionary procedure was taken for two main reasons: i) to prevent contamination of the air by highly allergenic pollen and ii) to prevent possible seed production and putative soil contamination of the garden. At the end of the summer season, the plant height corresponding to the distance between the top and the base of the plant was measured.

### Field study

To determine its ability to flower and to produce mature seeds in the northern part of the invaded European range, a total of 17 sites invaded by *A*. *artemisiifolia* were observed during the growing season 2010. Sites were located in and around two northern German localities; Drebkau (N 51°39´19”, E 14°13´25”) and Berlin (N 52°30´59”, E 13° 23´09”). The former locality was chosen because the population is known to be well established and distributed in the area, while the Berlin sites are probably infested with populations introduced at different occasions. Due to weed management measures during the growing season the number of sites was restricted, especially around Berlin where only four field and roadside sites were visited, with a minimum distance of 19 km between sites. To account for possible differences in selection pressure in the different habitats, due to e.g. time of harvest or control measures, six field sites and five roadside sites, respectively, were selected at Drebkau, with a minimum distance of 0.5 km between sites. The number of plants per site varied in most cases between 70 and 600 plants, but with two extremes of 3 000 and 15 000 plants. These latter two sites were transect-like stretched patches (50 m x 1 m and 20 m x 2 m, respectively, and the number of plants there was estimated by counting individuals per m^2^ and calculating for the area. Every week, 200 plants were randomly selected at each of these two sites for determination of the phenological stage. At all sites the phenological stages of individual plants were recorded weekly from the end of June until mid-October. Five successive stages of the development of the male inflorescence were pre-defined, recorded and estimated at site level: stage 0 = absence of male flower at the site, stage 1 = buds are visible, stage 2 = male flowers (start of pollen production), stage 3 = 80% of individuals at the site have mature male flowers (releasing pollen), stage 4 = male inflorescences dying. Four stages of the development of the female flower were pre-defined: stage 5 = absence of female flowers, stage 6 = female flowers (appearance of styles), stage 7 = 80% of the individuals have open female flowers (visible styles), stage 8 = 80% of the individuals have pollinated female flowers (swollen ovaries), while three stages of the fruit development were characterized: stage 9 = 80% of the individuals have maturing female flowers (ovaries at final seed size), stage 10 = 80% of the individuals have seeds with color changing from green to dark brown, stage 11 = mature seeds dispersing. Hence, the last stage of the female flower development corresponds to the stage before the start of seed maturation in the case of a successful pollination. The length of two important phenological stages were quantified: the “pollen production” stage (number of weeks between stage 2 and stage 4 of the development of male flowers), and the “total reproduction” stage (number of weeks between stage 1 of the development of male flower and stage 11 of the fruit development) (Table 2).

**Table 2:** Timing and length of phenology in field.

Phenological stage	Locality						Habitat at Drebkau					
	Drebkau		Berlin		S	P	Field		Roadside		S	P
	Mean±SE	n	Mean ±SE	n			Mean ±SE	n	Mean ±SE	n		
1. First bud	186.4 ±1.6	13	200.2 ±1.8	4	60.5	0.0048	189.2 ±2.8	6	185.2 ±1.7	5	24.5	0.3335
Pollen production												
2. Start	204.7 ±1.5	13	224.8 ±5.2	4	61.0	0.0037	206.7 ±3.0	6	202.0 ±0.0	5	22.5	0.1588
4. End	249.4 ±1.6	13	276.7 ±9.3	3	44.0	0.0101	247.5 ±1.6	6	251.0 ±3.8	5	33.0	0.6138
Female flowers												
6. Start	192.3 ±2.0	13	231.8 ±3.4	4	62.0	0.0030	196.2 ±3.3	6	188.0 ±3.8	5	23.0	0.2133
7. End	210.1 ±2.7	13	251.0 ±4.9	4	62.0	0.0030	210.2 ±3.3	6	213.2 ±5.7	5	31.5	0.8500
Fruit development												
8. Start	207.4 ±2.8	13	244.0 ±6.4	4	62.0	0.0036	210.2 ±3.3	6	209.0 ±4.9	5	29.0	0.9256
11. End	282.8 ±2.7	11	310.5 ±3.5	2	25.0	0.0320	277.6 ±3.4	5	286.0 ±4.9	4	25.5	0.1904
Length of stages												
Pollen production	45.2 ±2.2	13	56.0 ±8.1	3	35.5	0.1800	40.8 ±2.2	6	49 ±3.8	5	27.0	0.0993
Total reproduction	97.4 ±3.5	11	108.5 ±3.5	2	20.5	0.2245	89.6 ±3.4	5	101.5 ±6.1	4	26.0	0.1688

Differences in timing and length of the phenological stages of *A*. *artemisiifolia* plants recorded at two German localities (Drebkau and Berlin), and separately for two habitats in Drebkau (fields and roadsides). The timing of the phenological stages is given as Julian days, while the length of the phenological stages are presented as the number of days between the two stages defining it. The number for each phenological stage corresponds to the number given in the text for the different stages of male and female flowering. “n” denotes the number of sites. Values of S (test statistic associated with the smaller sample) and P (probability) were calculated using the Wilcoxon rank sum test.

### Statistics

Inter-population variations of male and female flowering time and plant height were analyzed by means of analysis of covariance, ANCOVA, using proc GLM in the statistical software SAS 9.3 (SAS Institute Inc. Cary, NC) with garden site as fixed factor, origin and the interaction as random factors. Since bird seed, which is the main vector for entrance of *Ambrosia* seeds to Sweden, may be imported from North America, it was considered important to include these populations in the analyses. The number of Julian days between the start of the garden experiment and the dates of first male or female flowering were calculated as mean for each population at each garden site. When analyzing the start of flowering, final plant height was included in the model to test for covariation, and when analyzing final plant height, seed weight was included in the model to test for covariation. For plant height, the partitioning of the variation between garden sites (environment) and among the different origins of the *Ambrosia* populations within site (genetic) was calculated using proc GLM.

To explore possible differences in the field observations between the two German localities, and between the different habitats (6 sites defined as field and 5 as roadside) within the locality of Drebkau, the non-parametric Wilcoxon sum rank test was used. The non-parametric test was used since most variables tested (first bud appearance, end of male flowering, and start and end of female flowering and fruit development) did not follow a normal distribution. The Julian days for a) appearance of buds, b) start and end of male flowering, c) start of female flowering and successful pollination (appearance of swollen ovaries) and d) start and end of seed maturation at each site was registered. In addition, the difference in length of the period of pollen production and the total reproduction period, calculated as the difference between the Julian days recorded for two phenological stages, was analyzed.

## Results

### Common garden experiment

**Fig 2:**
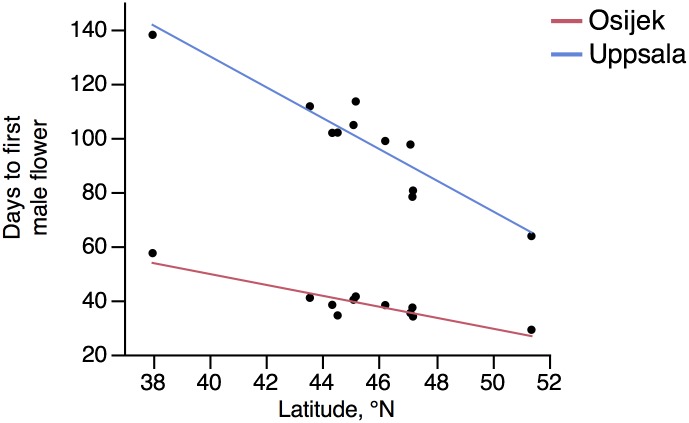
First male flowering of eleven *Ambrosia artemisiifolia* populations in a reciprocal common garden experiment. Gardens were located in Uppsala, Sweden (N 59° 48´ 55”, E 17° 38´ 47”) and in Osijek, Croatia (N 45° 31´ 16”, E 18° 40´ 54”). Mean number of days from start of common garden experiment until first male flower appearance of each population. Latitudes refer to sites from which seeds were collected (for details of populations and exact latitude information see Table 1).

**Fig 3:**
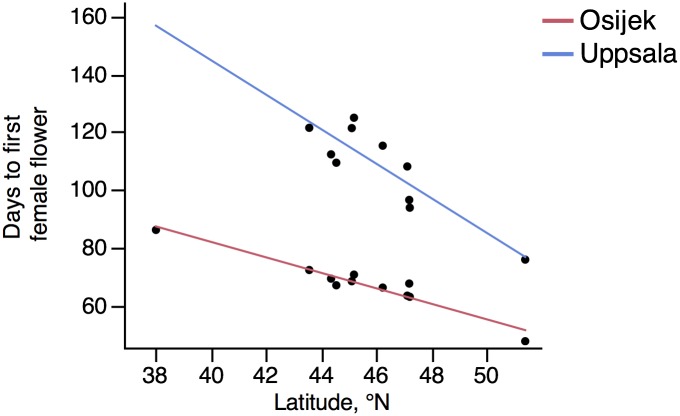
First female flowering of eleven *Ambrosia artemisiifolia* populations in a reciprocal common garden experiment. Gardens were located in Uppsala, Sweden (N 59° 48´ 55”, E 17° 38´ 47”) and in Osijek, Croatia (N 45° 31´ 16”, E 18° 40´ 54”). Mean number of days from start of common garden experiment until first female flower appearance of each population. Latitudes refer to sites from which seeds were collected (for details of populations and exact latitude information see Table 1). One population (Lexington) in Uppsala did not produce any female flowers.

The results revealed a clear north-south gradient for flowering time of *A*. *artemisiifolia* (Figs 2 and 3). Significant differences in the time required to trigger the male flowering and the female flowering were observed between populations within both gardens and also between the gardens, even if there were fewer populations producing female flowers in Uppsala than in Osijek (Table 3, Figs 2 and 3, S1 Appendix). Plants grown in Uppsala needed significantly more time to produce the first male and female flower than plants from the same populations grown in Osijek (Figs 2 and 3). Final plant height did not have a significant effect on either first male or female flower, hence the variable was excluded in the ANCOVA and only significant effects were stated in the final model. Early-flowering and late-flowering populations were identified as corresponding to the populations from the extreme northern and southern latitudes of our sampling (North: Germany 51.4°N; South: USA-Kt 38.0°N). In Osijek (Croatia) all the individuals, grown under good photo-thermic conditions (according to Deen and colleagues [34]), produced male and female flowers before the end of the common garden experiment. In contrast, at the site beyond the range limit (Uppsala, Sweden), 50% of the individual plants from the most southern population of our sampling (Lexington, Kentucky) failed to produce male flowers before onset of the Swedish frost. In total, approximately one fifth of the plants grown in Uppsala, representing four populations from the southern part of the invaded European range (southern France, Italy, Bosnia & Herzegovina) and the two American populations, did not produce female flowers (S1 Appendix).

**Table 3:** Analysis of covariance of latitude and common garden location on flowering time and plant height.

Source of variation	Male flower		Female flower^a^		Plant height	
	F^b^	P	F^b^	P	F^b^	P
Common garden	637.50	<0.0001	275.64	<0.0001	33.0	<0.0001
Latitude	101.20	<0.0001	65.16	<0.0001		
CG*Latitude	23.16	0.0001	11.04	0.0040		

*Ambrosia artemisiifolia* plants from 11 populations were grown in reciprocal common garden experiments in Uppsala, Sweden and in Osijek, Croatia. In the analysis of covariance first male flower, first female flower and final plant height were dependent variables with original latitude of sampled populations as the independent continuous variable and common garden site as the independent categorical variable.

^a^ The Lexington populations failed to produce female flowers when grown in Uppsala.

^b^ Degrees of freedom for the residual is 18, 17 and 18 for male and female flower and plant height, respectively.

**Fig 4:**
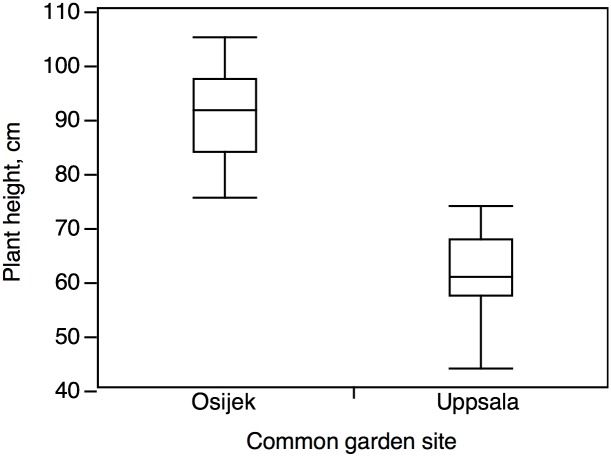
Plant height of eleven *Ambrosia artemisiifolia* populations in a reciprocal common garden experiment. Gardens were located in Uppsala, Sweden (N 59° 48´ 55”, E 17° 38´ 47”) and in Osijek, Croatia (N 45° 31´ 16”, E 18° 40´ 54”). Latitudes refer to sites from which seeds were collected (for details of populations and exact latitude information see Table 1).

Plants of different populations grown in Osijek were in general significantly taller (difference 27.6 cm, SE ±3.4) than plants of the same populations grown in Uppsala (Table 3, Fig 4). The effect of the origin of the population was not significant and represented only 14% of the variance of the final plant height (genetic effect “G”) while the difference between the two garden sites explained 75%. Seed weight did not have a significant effect on final plant height, hence the variable was excluded in the ANCOVA and only significant effects were stated in the final model.

### Field study

One of the German populations located in the northern part of the invaded European range (Drebkau) was also used in the common garden experiment. Phenological records from several sites confirmed its early-flowering in field conditions (middle of July; Table 2). Dates of the onset and ending of all phenological stages studied occurred significantly earlier at the sites in/around Drebkau (51°39”N) than at the sites in/around Berlin (52°30”N). However, no significant difference was observed for the length of the “pollen production” stage and of the “total reproduction” stage (Table 2) between these two separate German localities. No significant temporal difference was found among sites located in fields and sites situated along roads within the Drebkau locality for the start of the reproductive stage, the start and the end of male and female flowering, or the start and the end of the fruit development (Table 2).

## Discussion

Photoperiodic response has been proposed as one of the most important factors limiting the European distribution of invasive plants [12]. Attempts to predict the northward range shift of *A*. *artemisiifolia* have led to the conclusion that the extension of the range limit is to a large extent constrained by the photoperiod requirement for induction of flowering (e.g. [1, 4, 43]). However, our results indicate that the photoperiodic-thermal requirement is a trait with a large potential for local adaptation. Indeed, the results of the common garden experiment showed that *A*. *artemisiifolia* can form male and female flowers up to, at least, latitude 59°N, with a clear north-south gradient in flowering time. The large differences observed in the common garden experiment make it possible to differentiate early and late flowering populations and, thus, to estimate the potential threat of common ragweed populations to FennoScandinavian public health and agriculture (Figs 2 and 3).

The population representing the largest potential for establishment in FennoScandinavia, if introduced, is the most early-flowering population, originating from Germany. In contrast, the population from the southern part of the native range (Kentucky, USA) constitutes a negligible risk for invasion at north European latitudes. The German population is obviously well adapted to photoperiodic-thermal conditions at latitude 51°N, and our common garden experiment indicates that it is already pre-adapted for reproduction in northern Europe up to at least latitude 59°N. Thus, the niche limit of the German population seems to be beyond its present range limit.

It has been shown that the large ecological amplitude of *A*. *artemisiifolia*, together with unoccupied niches, is a factor behind successful invasion in France [22]. However, if a species has similar ecological and climatic niches in invasive and native populations, it has been seen that invasions typically fail without pre-adaptation. Under such circumstances, the rate of migration of suitable alleles along the selective gradient becomes crucial for adaptation [38]. Further, range expansion of a species is not only dependent on suitable intra specific ecological variation, available niche space and pre-adapted alleles, but also on successful establishment of locally adapted alleles. The latter is in turn influenced by gene flow, selection pressures and mutation rates [38]. In FennoScandinavia, the selection pressure on reproduction is rather strong, as only individuals with seeds set before the first frost will contribute to next generation. Such selection might counteract swamping of the locally adapted gene pool by pollen from central areas of the distribution range [38]. Several population genetic studies indicate that *A*. *artemisiifolia* in Europe has a high gene flow [27–29] and pollen from the central distribution range has been found in Sweden [44, 45]. In addition, seeds are dispersed by importation of *A*. *artemisiifolia* infected birdseed. Hence, from this perspective, the future establishment of viable populations of *A*. *artemisiifolia* in FennoScandinavia is a realistic scenario with strong selection pressure and the prerequisites for migration of pre-adapted alleles. This conclusion is supported by results from the German field study. Despite local adaptation at both localities, flowering occurred significantly earlier in Drebkau than in Berlin, which indicates that *A*. *artemisiifolia* has been introduced in northern Germany on several occasions.

To avoid exposing people in the area to allergenic pollen, individual male flower heads were cut at the first sign of pollen distribution at the Swedish common garden site, and thus it was not possible to record the date of seed setting. The early flowering of the German population at the northern site is, however, a clear indication that this population is pre-adapted to set seeds before frost at northern latitudes. Also, the dates of male and female flowering recorded at the northern common garden site coincided well with field observations of the Drebkau populations. The field observations at both German localities (Drebkau and Berlin) indicate that a period of 10–12 weeks after onset of female flowering is needed for seeds to mature (Table 2). Since the first female flowers of the German population at the Uppsala site were registered between 13 and 28 July we conclude that these individuals would have had ample time to produce mature seeds before the first autumn frost. In southern Sweden, the first frost occurs on average in the period 1 October to 1 November [46]. Corresponding dates for Drebkau are 11–20 October, and for Berlin 21–31 October [47].

Common garden experiments are widely used to investigate how environmental and genetic factors determine the success of invasive plants in their new non-native range. Moloney et al. [42] suggested a minimum optimal design (MOD) to increase the quality and utility of common garden experiments in invasion biology research. Their MOD includes multiple, strategic garden site locations, careful consideration of the genetic design, standardization of the experimental protocol, and care to ensure biosafety. In our experiment, sites were chosen to represent photoperiodic-thermal conditions both within and clearly beyond the present range limit of the species studied. Also, the populations included represented those close to the largest possible latitudinal range, and the experimental protocol was standardized for both sites. Further, biosafety precautions were taken at the northern site (non-invaded region) by cutting male flowers at the start of anthesis.

Environmental maternal effects have been shown to influence the growth of the offspring phenotype, thereby confusing the interpretation of a common garden experiment [48]. The effects are most pronounced on traits early in development of the offspring, i.e. dormancy, germination and seedling growth. The maternal effect diminishes over time, and the effect on the adult plant is in most cases an indirect effect of seed size [49, 50]. Moloney et al. [42] recommended including plant height at transplanting as a covariate in the statistical analysis. To compensate for possible maternal effects, we used seedlings of the same size for transplanting, and statistical analysis revealed no significant effect of seed weight on final plant height in either Uppsala or Osijek (S2 Appendix). In addition, plant height, in contrast to flowering time, was not significantly affected by latitude. Both results support our conclusion that response to photoperiodic-thermal conditions can be attributed to adaptive variation, and not to environmental maternal effects.

The large variation in phenological responses of the locally adapted *A*. *artemisiifolia* populations investigated demonstrates very clearly the need for integrating this local adaptation in forecasts of future range shifts. Recent works on species distribution or pollen dispersal models for *A*. *artemisiifolia* [1, 4, 51, 52] have used phenological data produced by only two populations from the native range (Canada, 43.06°N in [31]; USA, 42.13°N in [53]). These models do not integrate the phenological variations of the different introduced, established and locally adapted European populations. This might explain the incongruity between the predicted absence of successful reproductions at latitudes higher than 55°N within the species distribution model [4] and the early flowering of the German population observed here, in our garden experiment at latitude 59°N. According to the model, the range limit of *A*. *artemisiifolia* will not have reached latitude 60°N until 2050. We conclude that, to improve species distribution models, molecular phylogeographical investigations should be done on large and representative numbers of native and introduced populations to group them in different genotypic clusters. Subsequently, common garden or growth chamber experiments could be done to differentiate these genotypic clusters depending on their physiological responses and improve the model by sharpening its settings with their specific parameters.

The latitudinal north-south gradient in common ragweed is more observable and distinguishable under non-optimal photoperiodic-thermal conditions than under more optimal conditions. This emphasizes the need for more studies involving common garden experiments beyond the studied species´ range. Earlier reports show a negative correlation between maximum aboveground biomass and latitude [16], which indicates that there exists a trade-off between early flowering and plant height. However, here plant height was significantly affected by the common garden sites but not by the latitudinal origin of populations. To enable good predictions regarding establishment, studies on reproduction and possible trade-offs between phenological traits (timely seed set) and biomass (seed number, competitive effects) are needed. These should be conducted using common garden experiments at more than one site, situated both within and beyond the range limit, i.e. also in a potential invasion area. Phenological north-south gradients, similar to our findings, have been revealed earlier [16, 54, 55] in common ragweed. However, none of these studies were done at more than one common garden experiment site, and only at sites within the species´ range of distribution.

To conclude, there exists a high risk for establishment and spread of *A*. *artemisiifolia* in FennoScandinavia in the near future. The range shift may be enhanced by climate change resulting in an extended growing season [56] but this is not a prerequisite. As stated by Clements and DiTomasso [39], evolution might lead to faster and larger range expansion than earlier anticipated, something that has been exemplified here with the German *A*. *artemisiifolia* populations pre-adapted to northern latitudes.

## Supporting Information

S1 Appendix Number of Julian days to first male and female flower of *Ambrosia artemisiifolia* in a reciprocal common garden experiment. (TIF)

S2 Appendix Correlation of plant height and seed weight of *Ambrosia artemisiifolia* in a reciprocal common garden experiment. (TIFF)
