# Supplementary figures and images for: Phenological Variation in Ambrosia artemisiifolia L. Facilitates Near Future Establishment at Northern Latitudes
Source: PLoS One. 2016 Nov 15;11(11):e0166510. doi: 10.1371/journal.pone.0166510 (PMC5113013; doi:10.1371/journal.pone.0166510)

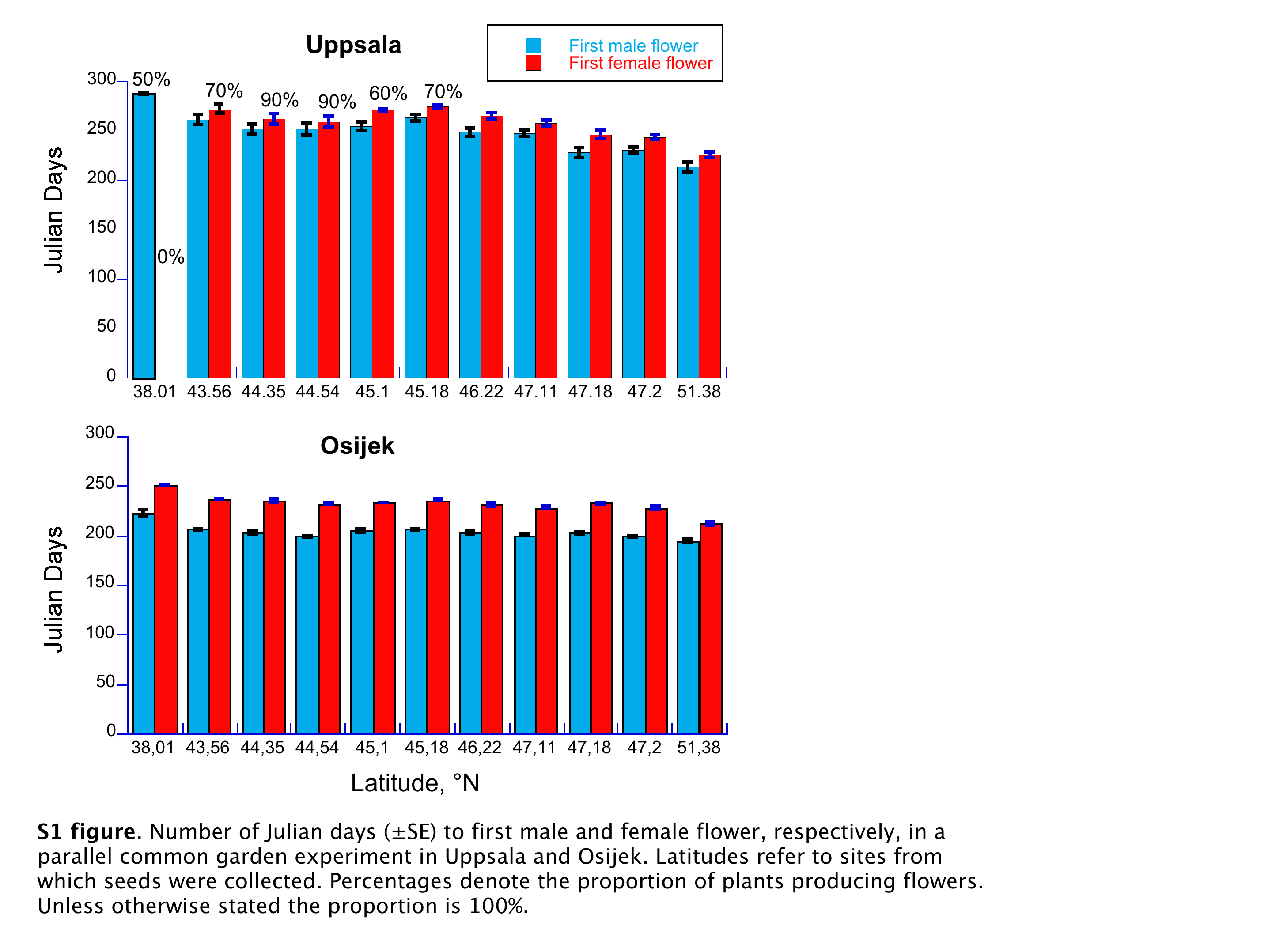

Supplement: S1 Appendix — (TIF) [file pone.0166510.s001.tif]

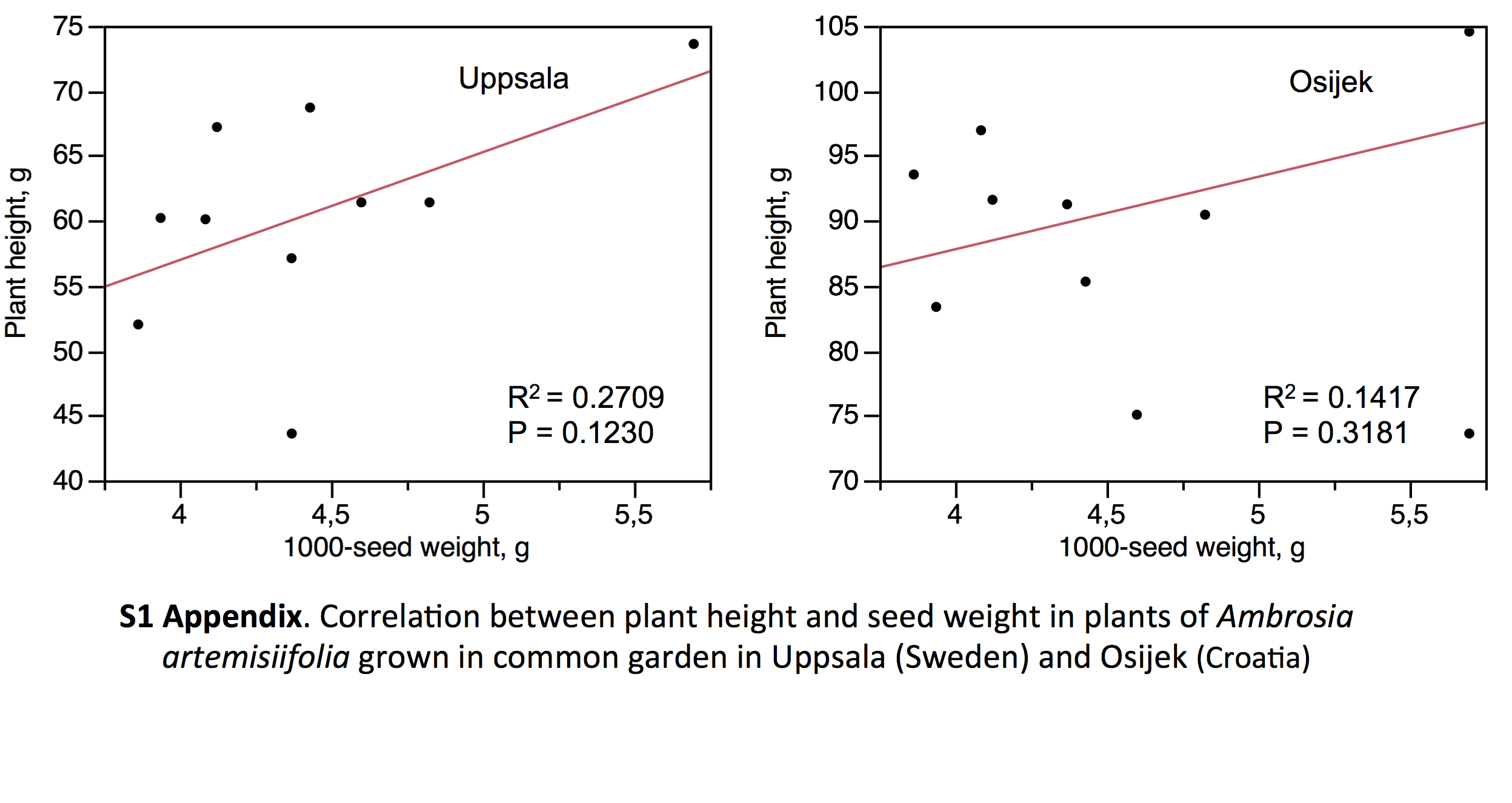

Supplement: S2 Appendix — (TIFF) [file pone.0166510.s002.tiff]
